# Teaching trainees how to critically evaluate the literature - a crossover study at two pediatric residency programs

**DOI:** 10.5116/ijme.58ce.5f04

**Published:** 2017-04-24

**Authors:** Benjamin Nelson, Catherine Ingard, David Nelson

**Affiliations:** 1 Department of Pediatric Pulmonology, Massachusetts General Hospital for Children, Boston, MA, USA; 2 Department of Pediatrics, MedStar Georgetown University Hospital, Washington, DC, USA

**Keywords:** Graduate medical education, evidence based medicine, pediatric residents, USA

## Abstract

**Objectives:**

The purpose of this study was to assess the efficacy of a concise, evidence based medicine curriculum in improving the knowledge of pediatric residents at two institutions.

**Methods:**

Sixty first and second year pediatric residents at MassGeneral Hospital for Children and MedStar Georgetown University Hospital participated in a crossover study. The evidence based medicine curriculum, consisting of 4 ninety minute sessions grounded in adult learning theory principles, was developed using the methodology described in the book ‘Studying a Study’. A validated 20 question evidence based medicine multiple choice test was administered on three separate occasions to measure baseline knowledge, efficacy of the curriculum in improving knowledge, and long term retention of that knowledge.

**Results:**

Post curriculum, the fall group’s scores improved 23% from baseline (M=10.3, SD=2.4) to (M=12.7, SD=3.0) students (t_(26)_=-3.29, p=0.0018) while the spring group improved by 41% (M=10.0, SD=2.8) to (M=14.1, SD=2.2) students (t_(32)_=-6.46, p<0.0001).  When re-tested 4-6 months later, the fall group’s scores did not significantly decline from their immediate post curriculum scores (M=12.7, SD=3.0) to (M=11.7, SD=3.0) students (t_(32)_ =1.33, p=0.190). There was an association between number of sessions attended and increase in post curriculum score (χ^2^(3, N=60) =11.75, p=0.0083).

**Conclusions:**

Findings demonstrate our curriculum was effective in teaching evidence based medicine to pediatric residents, and fostered long term retention of knowledge.  Based on these results, we believe this curriculum could be implemented at any institution.

## Introduction

Understanding the concepts of evidence based medicine (EBM) with the ability to assess the medical literature is a necessary skill for all clinicians.  However, most residents have limited knowledge of the components of research methodology including study design, biostatistics, and result interpretation.^1^ Any knowledge acquired appears to regress with advancement through training, possibly due to the instruction taking place during the undergraduate medical education years with limited reinforcement during residency.  Although many pediatric programs offer an EBM curriculum in some form, a survey of chief residents at North American residency programs found that 7% (n=153) felt confident in their ability to teach EBM, and only 20% (n=153) were able to evaluate EBM effectiveness.^2^

Given the time constraints on didactic instruction during residency, it is difficult to introduce new curriculum.  Therefore, journal club is the most commonly used vehicle to teach EBM to residents, yet this rarely translates into effective EBM skills.^2^ Basing curriculum on adult learning theory is critical^3^ in engaging adult learners and fostering knowledge retention, but these principles are rarely applied in the context of these journal clubs.  Due to logistical issues with residents’ schedules, and lack of protected time for EBM curricula, few studies have been able to assess the effectiveness of EBM training.^4^^,^^5^  Therefore, most studies assess trainee’s knowledge immediately after a course or intervention but have not routinely measured knowledge retention months later.

Past research has shown that short courses which provide six hours of instruction are sufficient to improve pediatric residents’ EBM skills,^6^  however, very little is known about either the generalizability or long term effectiveness of these courses.  Studies that have assessed EBM effectiveness have either looked at courses spanning several years of training,^7^^,^^8^ or have only measured self-reported outcomes.^9^ None have been carried out at more than one institution.

Currently, it is unknown if a concise EBM course grounded in adult learning theory, could achieve long term effectiveness and be generalizable to other institutions.  The purpose of this study was to assess both the short and long term effectiveness of a novel EBM curriculum in improving the knowledge of pediatric residents at two institutions.

We hypothesized that pediatric residents’ current understanding of EBM is limited, and that our curriculum, grounded in adult learning theory, would improve pediatric residents’ understanding of study methodology, biostatistics and result interpretation, leading to long term retention and improved confidence in understanding these principles.

## Methods

### Study design and participants

The IRBs at each institution, MassGeneral Hospital for Children (MGHfC) and MedStar Georgetown University Hospital (MGUH) determined this study exempt from ethical approval as the research was conducted in established or commonly accepted educational settings, involved normal educational practices and examined the effectiveness of instructional techniques.

Participants included first and second year residents at two pediatric residency programs, MGHfC and MGUH. All MGHfC residents were eligible to participate. Eligible MGUH residents were on campus for clinical rotations during the study.  Residents were split into fall and spring groups.  The fall group consisted of first and second year MGUH residents and all second year residents at MGHfC.  The spring group consisted of first and second year MGUH residents and all first year residents at MGHfC.  MGUH residents were assigned to the fall or spring group based on rotation schedule.  All residents were made aware of the study, with participation strictly voluntary. 

Sixty-three residents were eligible to participate in this study, 46 from MGHfC and 17 from MGUH; 95% (n=63) of eligible residents were included with no dropouts.  Baseline characteristics including age, holding an advanced degree, and prior EBM training were similar between institutions; 93% (n=46) of MGHfC residents and 82% (n=17) of MGUH residents had previously taken a course in epidemiology, biostatistics or EBM.

A crossover methodology where all residents eventually completed the curriculum was used.  Prior to the start of the study, all residents were tested to determine their baseline EBM knowledge. The fall group participated in the new EBM curriculum, while the spring group, serving as controls, participated in regularly scheduled residency activities. At the end of the fall curriculum, both groups were again tested on their EBM knowledge.  The changes in scores from baseline would reflect the effect of the intervention. Subsequently, the spring group participated in the same curriculum, while the fall group participated in regularly scheduled residency activities.  At the completion of the spring curriculum, both groups were tested for the third time.  The spring group’s change in scores reflected efficacy of the curriculum, while the fall group’s scores (measured 4-6 months after completing the curriculum) were used to measure knowledge retention.

### Curriculum

We developed an EBM curriculum focused on critically evaluating the clinical literature.  Sessions were developed using the systematic methodology described in Studying a Study.^10^ The MAARIE (Method, Assignment, Assessment, Results, Interpretation, Extrapolation) framework was used as the systematic appraisal instrument. The framework allows any study, regardless of methodology, to be assessed on the above six components.

The curriculum consisted of 4 ninety minute sessions given during existing blocked teaching time (MGHfC) or separate evening sessions (MGUH).  At a given institution, all sessions were led by one of the authors (BAN and DBN).  Only the Georgetown instructor had prior epidemiological training. Both instructors used the approach outlined in the book, Studying a Study and Testing a Test,^10^ to prepare for each session.

The four sessions built on one another and were based on adult learning theory principles emphasizing experiential learning.  Content included didactic sessions, flaw catching exercises, multiple choice questions using audience response systems, and interactive review of abstracts and articles. The articles and abstracts were evaluated using the MAARIE framework. The curriculum was identical at each institution (Table 1).

### Data collection methods

A modified intention to treat analysis was used.  Residents had to attend at least one session and complete the pre-test to be included.  Three residents were excluded: one resident from each institution did not attend any sessions, and 1 did not complete the pre-test. 

Performance improvement on a biostatistics and study design multiple choice test was the primary outcome measure. This test has been shown to have content and discriminative validity with internal consistency assessing understanding of commonly used statistics, study design, and interpretation of study results.^1^ The test consisted of 20 multiple choice questions, each worth 1 point.  Questions were clinically oriented with a case vignette, and did not require calculations. Questions addressed statistical methods, confidence intervals, p values, sensitivity and specificity, power and sample size, study design, and interpretation of study results.  The test was administered three separate times to the participants.  The same test questions were used each time, but the order varied to limit learning effect.  Residents were provided the answers only after the study was complete.

**Table 1:** Description of the EBM curriculum (each session was 90 minutes)

EBM curriculum	Session 1	Session 2	Session 3	Session 4
In class	EBM test	Review multiple choice questions in teams using audience response system	Multiple choice questions covering session 2 information (small groups)	Review of MAARIE framework and key concepts
	Flaw Catching exercise	Introduction to MAARIE framework (2^nd^ half R.I.E.)	Apply MAARIE framework to abstracts	Apply MAARIE framework to RCT #2
	Introduction to MAARIE framework (1^st^ half M.A.A.)	Flaw catching exercise	Apply MAARIE framework to RCT #1	EBM test
Homework	Multiple choice questions covering session 1 information	Read randomized controlled trial (RCT) #1	Read RCT #2	

Using a 5 point Likert scale anchored with no confidence (1) and complete confidence (5), residents were asked to rate their confidence in interpreting commonly used statistical tests before and after participating in the EBM curriculum. These included their ability to interpret p values and other statistical measures, determining if the correct statistical procedure was used, and identifying factors that relate to a study’s power.  At the completion of the course, residents were also asked to rate their experience using a 5 point Likert scale (1=poor, 5=great). They were asked their level of overall enjoyment, to assess the relevance of the course, if they learned skills which they could apply to daily practice, and whether they would recommend the course to their peers.

### Statistical analysis

Paired t-tests were used for comparisons between pre and post intervention test scores. The Kruskal-Wallis test was used to determine if number of sessions attended affected performance on the post test. A symmetry test, an extension of the McNemar paired proportions test for multi-category variables, was used for the Likert data to examine changes in the responses as a result of the intervention.  A p value < 0.05 indicated statistical significance.

## Results

**Figure 1:**
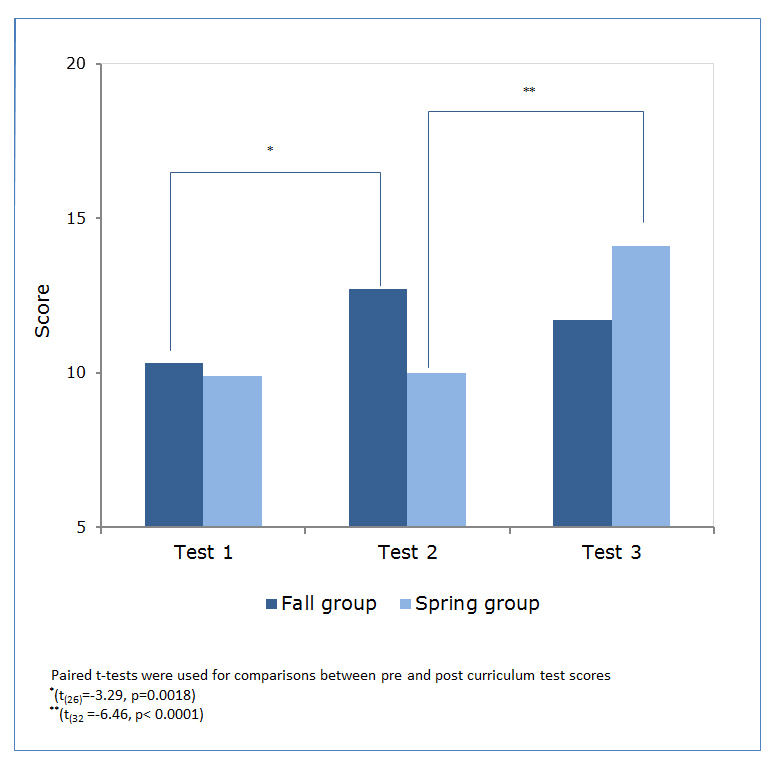
Pediatric resident test scores pre and post curriculum

Baseline (test 1) scores were similar between the two groups (Figure 1). After completing the curriculum, the fall group’s score (out of 20) improved 23% from baseline (M=10.3, SD=2.4) to (M=12.7, SD=3.0) students (t_(__26)_=-3.29, p=0.0018) while the spring (control) group, participating in regularly scheduled residency activities, remained constant (M=9.9, SD=2.9) to (M=10.0, SD=2.8) (test 2). The spring group did show improvement (test 3), however, from their baseline scores after completing the curriculum.  Their score (out of 20) improved by 41% (M=10.0, SD=2.8) to (M=14.1, SD=2.2) students (t_(__32)_=-6.46, p< 0.0001).  The fall group’s scores (test 3), measured 4-6 months after completing the curriculum, did not significantly decline from their immediate post test scores, demonstrating retention of the knowledge gained from their original participation (M=12.7, SD=3.0) to (M=11.7, SD=3.0) students (t_(32)_ = 1.33 p=0.190).

**Figure 2:**
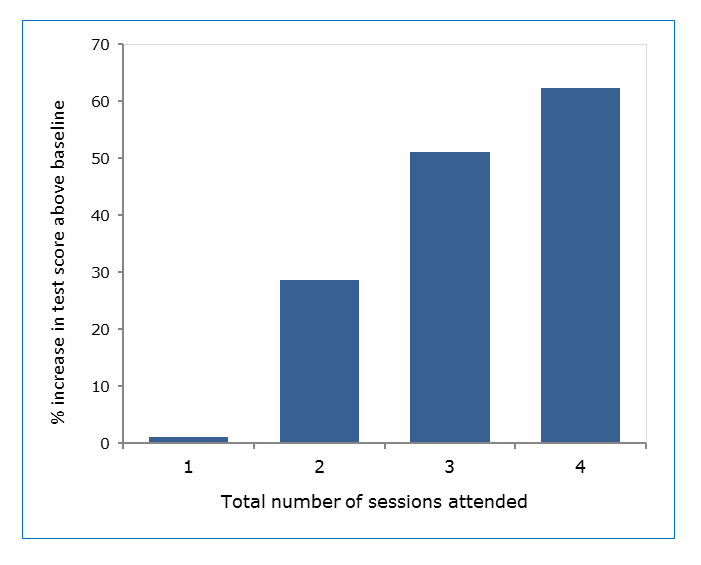
Number of sessions attended is associated with an increase in test score above baseline

Five residents (8%) attended only one session, 17 (28%) attended 2 sessions, 28 (47%) attended 3 sessions, and 10 (17%) attended all 4 sessions. A Kruskal-Wallis test was performed to explore the association between number of sessions attended and increase in baseline test score. There was an association (χ^2^ (3, N=60) = 11.75, p= 0.0083) between number of sessions attended and improvement in test scores. Those who attended four sessions improved by 60% over baseline compared to 30% for those who attended only two sessions (Figure 2).

Upon completion of the course, residents rated their experience as well as their confidence in interpreting commonly used statistical tests.  Rating their experience on a five point scale, they enjoyed the course (M=4.1), found it extremely relevant (M=4.6), felt they learned skills they could apply to their daily practice (M=4.4), and would recommend this course to their peers (M=4.5).  Residents’ confidence at both institutions showed statistically significant improvement post curriculum in their ability to assess if the correct statistical procedure was used (χ^2^ (4, N=60) =31.5, p=0.0005), and in identifying factors that influence a study’s power (χ^2^ (4, N=60) =25.8, p=0.004). Although not reaching statistical significance, more residents felt confident in interpreting the p-value for a given result and interpreting the results of a statistical method.

## Discussion

The introduction of a critical appraisal tool, the MAARIE framework,^10^ taught in the context of a short six hour curriculum, substantially improved residents’ knowledge and confidence when analyzing the clinical literature. This was accomplished without committing substantial resources, and did not require faculty with prior EBM experience. 

Similar to previous studies,^1^ we showed that pediatric residents have limited EBM baseline knowledge.  Unlike prior studies measuring effectiveness of EBM courses,^4^^-^^9^ we showed both a substantial improvement in knowledge acquisition, and demonstrated long term retention among residents with varying levels of training.  We were able to do this at two uniquely different institutions.

To improve knowledge transfer, we applied Kolb’s experiential learning model to our curriculum design^11^ and incorporated adult learning principles such as spaced learning, interleaving, and desirable difficulties.  We believe this model engaged our learners, and contributed to higher satisfaction ratings leading to improved knowledge retention. 

Both the fall and spring groups showed a statistically significant increase in test scores above baseline after participating in the EBM curriculum. The instructors’ increased familiarity with the course and initial feedback from the residents may explain the greater improvement in test scores seen in the spring group.  Pre and post curriculum confidence scores did not vary between the fall and spring groups, suggesting that each group had a similar educational experience.

The strength of our study was the ability to demonstrate knowledge retention 4-6 months after completing the curriculum. Furthermore, simply participating in a pediatric residency did not teach these important skills, arguing against a maturation effect. We found a statistically significant dose response in our study; attending more sessions was associated with a greater improvement in post curriculum test score. When analyzing each institution separately, MGUH residents showed slightly more improvement in post intervention scores than MGHfC, which may be explained by a higher attendance rate. This highlights the importance of finding time in the residency schedule to allow full participation.  We demonstrated evening dinner sessions are feasible, and survey results showed that evening sessions did not negatively affect the residents’ perception of the course.

Residents at both institutions rated their experience favorably, felt more confident interpreting statistical tests post curriculum, and recommended that the course be repeated. This study suggests that a short four session course using a critical appraisal instrument can serve as the basis for an effective and rigorous EBM curriculum.  There was nothing inherent in the curriculum that would limit it to only pediatric residents. All examples and articles included in the curriculum can be changed to be specialty specific, and no prior knowledge is necessary to understand the material.  Therefore, this course could be used for all specialties regardless of level of training, or prior EBM experience.

### Study limitations

The residency programs at MGHfC and MGUH were different sizes and because of logistical issues first year residents were grouped with second year residents at only one institution.  Since all residents in the study were similar in background demographics and baseline EBM knowledge, it was appropriate to combine them.  The mixing of groups may actually improve the generalizability of our results and when analyzed separately, scores at each individual institution were similar. Finding protected time in the residents’ schedule was challenging.  Therefore, some residents could not attend all sessions, and the sessions did not occur at equally spaced intervals at the two institutions. Since this is likely to happen in actual practice, it gave us the opportunity to see if this curriculum would work in a real life situation. Although the content was identical, and every effort was made to deliver the material in a similar fashion, it is probable the residents received somewhat different instruction at each institution. However, MGHfC residents showed similar improvement to the MGUH residents despite only the MGUH instructor having previous epidemiological training. The primary outcome measure was administered three separate times and used the same questions, however, the order varied and residents were not given the answers until completion of the study.  Simply taking the test multiple times did not improve residents’ scores and no testing effect was seen.

While this study did not measure changes in behavior, residents were encouraged to use the MAARIE framework on their own and in the setting of existing journal clubs.  Future studies will determine if sustained use of the MAARIE framework will continue to improve pediatric residents’ knowledge and skills, and ultimately lead to applying EBM skills to patient care.

In the future we intend to offer this curriculum at the beginning of intern year, followed by regular journal clubs based on the MAARIE framework. We have shown this curriculum to be effective when led by faculty without EBM training, and plan to teach other faculty to deliver this course.  We will continue to study long term retention using the MAARIE framework in future journal clubs. 

## Conclusions

Effective curricula need to be adult learning focused, promote self-directed learning, fill an educational gap, fit into a busy training schedule and be cost effective. This curriculum satisfies these requirements, empowering the trainee to critically appraise the literature in a more confident manner, and provides a foundation for future learning.

This study depicts a novel, effective approach to teaching EBM to pediatric residents which improves knowledge and fosters long term retention. We believe this curriculum could be implemented at any institution, and should be applicable across subspecialties for students with varying levels of prior knowledge.

### Acknowledgements

The authors would like to thank the Department of Biostatistics and Bioinformatics, MedStar Health Research Institute for providing statistical support.

### Conflict of Interest

The authors declare that they have no conflict of interest.
